# 286 A Comparison of Teleburn to In-person Consultations of Pediatric Patients in a Children’s Emergency Department

**DOI:** 10.1093/jbcr/irad045.261

**Published:** 2023-08-29

**Authors:** Claci Ayers, Hannah Byrd, Stephen Gondek, Rebecca Kidd, Ronnie Mubang, Barbara J Solomon, Anne L Wagner

**Affiliations:** Vanderbilt University Medical Center, Nashville, Tennessee; Vanderbilt University Medical Center - Monroe Carell Jr. Children’s Hospital, Nashville, Tennessee; Vanderbilt University Medical Center, Nashville, Tennessee; Monroe Carell Jr Children’s Hospital at Vanderbilt, Nashville, Tennessee; Vanderbilt University Medical Center, Nashville, Tennessee; Monroe Carell Jr. Children’s Hospital at Vanderbilt - - Nashville, TN, Nashville, Tennessee; Vanderbilt University Medical Center, Nashville, Tennessee

## Abstract

**Introduction:**

Regional Burn Centers provide burn care but there are disparities in access to regional burn centers for pediatric patients. Teleburn is a tool that enables providers without a certified burn center to provide photos of a burn to experts and receive recommendations on burn care. Our facility implemented a Teleburn system in December 2019. The purpose of this study is to evaluate the effectiveness of a Teleburn system to the gold standard in-person consultation in regards to burn infection rate, clinic follow up rate, post-burn admission rate, and 72-hour bounce back rate.

**Methods:**

Data was collected from December 2019-March 2022 through the electronic medical record. A total of 416 patient encounters that met criteria were analyzed. The criteria for a Teleburn encounter are displayed in Fig. 1 and this was the criteria applied to the in-person burn consults. A non-inferiority study was designed comparing proportional outcomes of telemedicine burn initial visits to emergency department visits in regard to burn infection rate, clinic follow up rate, post-burn admission rate, and 72-hour bounce back rate. The data were compared with a difference of greater than 10% being considered inferior.

**Results:**

Amongst the patient encounters 45.6% were female and ages ranged from 7 weeks to 19 years. Most common burn type was scald at 47.5%, with thermal being second at 43.7%. No differences were identified in rates of readmission - 1.67% difference (95% CI -27%< x< 23.8%) and return within 72 hours – 0.7% difference (-18.4%< x< 19.7%). Teleburn patients were 12.6% less likely to follow up (2.7%< x< 22.40%). Only one infection was identified, which is insufficient to conclude non-inferiority in terms of infection events.

**Conclusions:**

While convenient, Teleburn consult could not be demonstrated to be non-inferior to in-person consultation. No differences in infection rates were identified, and difference in readmission and return were clinically insignificant. Efforts to improve follow-up may prove successful in raising the standard to that of in person visits given the large geographic areas covered by most regional burn centers.

**Applicability of Research to Practice:**

This research advocates for more enhancement of Teleburn consult systems for pediatric acute minor burns and presents easily surmountable limitations. This study demonstrates that telemedicine may be effective and feasible to regional burn centers if follow up can be improved.

